# Reactive Centre Loop Mutagenesis of SerpinB3 to Target TMPRSS2 and Furin: Inhibition of SARS-CoV-2 Cell Entry and Replication

**DOI:** 10.3390/ijms232012522

**Published:** 2022-10-19

**Authors:** Saravjeet Singh, Sophie O’Reilly, Hossam Gewaid, Andrew G. Bowie, Virginie Gautier, D. Margaret Worrall

**Affiliations:** 1 School of Biomolecular and Biomedical Science, University College Dublin, Belfield, D04 V1W8 Dublin, Ireland; 2 UCD Centre for Experimental Pathogen Host Research (CEPHR), School of Medicine, University College Dublin, Belfield, D04 V1W8 Dublin, Ireland; 3 The School of Biochemistry and Immunology, Trinity Biomedical Sciences Institute, Trinity College Dublin, D02 R590 Dublin, Ireland

**Keywords:** SARS-CoV-2, SerpinB3, TMPRSS2, furin, cathepsin L, antiviral, designer serpin

## Abstract

The SARS-CoV-2 virus can utilize host cell proteases to facilitate cell entry, whereby the Spike (S) protein is cleaved at two specific sites to enable membrane fusion. Furin, transmembrane protease serine 2 (TMPRSS2), and cathepsin L (CatL) are the major proteases implicated, and are thus targets for anti-viral therapy. The human serpin (serine protease inhibitor) alpha-1 antitrypsin (A1AT) shows inhibitory activity for TMPRSS2, and has previously been found to suppress cell infection with SARS-CoV-2. Here, we have generated modified serpin inhibitors with increased specificity for these cellular proteases. Using SerpinB3 (SCCA-1), a cross-class inhibitor of CatL, as a scaffold, we have designed and produced reactive centre loop (RCL) variants to more specifically target both furin and TMPRSS2. Two further variants were generated by substituting the RCL P7–P1 with the spike protein S1/S2 cleavage site from either SARS-CoV-2 alpha or delta (P681R) sequences. Altered inhibitory specificity of purified recombinant proteins was verified in protease assays, with attenuated CatL inhibition and gain of furin or TMPRSS2 inhibition, as predicted, and modified serpins were shown to block S protein cleavage in vitro. Furthermore, the serpin variants were able to inhibit S-pseudoparticle entry into A549-ACE2-TMPRSS2 cells and suppress SARS-CoV-2 replication in Vero E6 cells expressing TMPRSS2. The construct designed to inhibit TMPRSS2 (B3-TMP) was most potent. It was more effective than A1AT for TMPRSS2 enzyme inhibition (with an eighteen-fold improvement in the second order inhibition rate constant) and for blocking SARS-CoV-2 viral replication. These findings advance the potential for serpin RCL mutagenesis to generate new inhibitors, and may lead to novel anti-viral biological molecules.

## 1. Introduction

**Figure 1:**
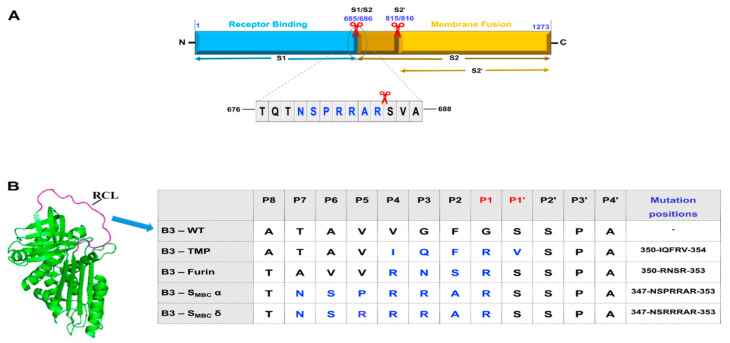
SerpinB3 variants designed with modified RCL sequences to inhibit TMPRSS2 and furin. (**A**) Schematic representation of coronavirus spike protein with cleavage sites. (**B**) The structure of native serpin archetype with RCL highlighted in magenta, containing a protease cleavage site (P1-P1’). Amino acid substitutions are highlighted in blue in the table, with the SerpinB3-WT sequence shown in black. The image was made in PyMol using the PDB file code 1QLP.

There has been rapid progress on the understanding of SARS-CoV-2 mechanisms of infection following the onset of the COVID-19 pandemic [1] along with unprecedented efforts to develop therapeutic strategies for prevention and treatment [2]. The entry of the virus into host cells is dependent on the major surface protein, the spike (S) protein, which facilitates angiotensin-converting enzyme (ACE2) receptor binding and fusion of the lipid envelope with host cell membranes. The S protein has two distinct functional domains, and cleavage by host cell proteolytic enzymes is required for membrane fusion and cell entry, as informed by previous work on coronavirus and influenza infection [3,4]. Two cleavage sites have been identified: an S1/S2 site containing a unique multibasic motif not found in other coronaviruses, with the sequence PRRARS and cleavage between amino acids 685–686 [5,6]; and an S2’ cleavage site at 815–816, which is key for facilitating membrane fusion (Figure 1A). Loss of the multibasic or furin cleavage site attenuated SARS-CoV-2 pathogenesis in both hamster and mouse models of infectivity [7]. A synthetic furin inhibitor, MI-1851, strongly inhibited SARS-CoV-2 replication in human airway cells [8], although furin knockout showed reduced rather than abolished S1/S2 cleavage, suggesting that while furin promotes infectivity, it is not essential for it [9]. Enhanced cleavage for the delta SARS-CoV-2 variant can be attributed to a mutation (P681R) at this spike protein site [10].

For S2’ cleavage TMPRSS2 is the major host protease thought to be involved [11]. TMPRSS2 is a type II transmembrane serine protease expressed in epithelial cells, e.g., in respiratory and prostate tissues, and known to activate fusion-facilitating glycoproteins in other viruses [12]. In VeroE6 cells, overexpression of TMPRSS2 can increase SARS-CoV-2 viral replication 100-fold by promoting membrane fusion at the cell surface [13]. TMPRSS2 is upregulated by inflammatory cytokines, with evidence that IL1β promotes TMPRSS2 expression via the p38 MAPK-GATA2 axis, enhancing SARS-CoV-2 entry in human lung cells [14].

An alternate mechanism of viral entry via endocytosis occurs whereby endosomal proteases cleave the spike protein, with cathepsin L (CatL) involvement a key factor. Koch et al. demonstrated that TMPRSS2 expression dictates the mechanism of entry; SARS-CoV-2 viral entry was rapid (within 10 min) in the presence of TMPRSS2, however, for target cells lacking TMPRSS2, a pH dependent endocytosis with cathepsin-mediated cleavage was evidenced as a longer 40–60 min entry route [15].

Because host proteases control initial entry of SARS-CoV-2 into host cells, and thus contribute to initiation of disease, these enzymes have become an important therapeutic target in COVID-19 [16]. Unlike inhibitors that block the viral encoded main M-Pro protease, host cell surface protease inhibitors may have broader antiviral potential for both influenza and coronavirus infections [4]. Candidate small molecule inhibitors with promising activity include the furin inhibitor MI-1851 [8] and the TMPRSS2 inhibitor camostat mesylate [11]. More recently, two promising ketobenzothiazole inhibitors of TMPRSS2, namely, MM3122 [17] and N-0385 [18], have been advanced for clinical development.

Naturally occurring protease inhibitors have therapeutic potential as well; serpins are the major human class of protease inhibitors, using a suicide mechanism via an exposed Reactive Centre Loop (RCL) that acts as a pseudosubstrate to target serine and cysteine proteases. Selectivity for target proteases is largely determined by the amino acid residues flanking the RCL cleavage site. Previous studies have shown that alpha-1 antitrypsin (A1AT, SerpinA1) can inhibit cell-based cleavage of the fluorescent TMPRSS2 substrate Boc-QAR-AMC [19] and can inhibit recombinant TMPRSS2 [20]. In addition, Prolastin, a therapeutic A1AT preparation, can block SARS-CoV-2 infection in cells, including human airway epithelial cultures [20]. However, A1AT is known to inhibit a broad range of human proteases, including neutrophil elastase, trypsin, kallikreins, and cathepsin G [21].

In this study, we set out to modify the serpin RCL in order to generate variants with improved specificity towards either furin or TMPRSS2. We used recombinant SerpinB3, an endogenous inhibitor of the endosomal protease CatL (one of our three SARS-CoV-2 antiviral targets), as a scaffold for rational design of specific variants. The RCL sequence was altered to match known subsite specificities of furin and TMPRSS2 or to match the unique multibasic sequence in the SARS-CoV-2 spike protein, with between four and seven amino acids replaced. The expected change in inhibitory profiles was demonstrated in fluorometric substrate cleavage assays, and the wild-type and modified serpins could block S protein cleavage by the individual target protease in vitro. Furthermore, the recombinant proteins inhibited both SARS-CoV-2 S pseudoparticle entry into A549 cells expressing ACE2 and TMPRSS2 and suppressed SARS-CoV-2 replication in TMPRSS2 expressing VeroE6 cells, with the anti-TMPRSS2 designer serpin (B3-TMP) showing the best efficacy and greater potency than A1AT.

## 2. Results

### 2.1. Designer SerpinB3—RCL Mutagenesis

To design novel inhibitors to block SARS-CoV-2 cell entry, our template recombinant serpin was SerpinB3 (or SCCA-1), a 44.5 kDa cross-class inhibitor of cysteine proteases, including the target, CatL. However, the closely related SerpinB4 (SCCA-2) inhibits the serine proteases chymotrypsin and cathepsin G, and Schick et al. have shown that RCL loop swaps are able to change the specificity of B3 or B4 effectively [22]. Therefore, the SerpinB3 backbone is not protease-class specific, and can be an effective template scaffold to target proteases with either serine or cysteine active site nucleophiles.

Three approaches were used to design potential inhibitors of SARS-CoV-2 infection (Figure 1B). First, a construct was designed to inhibit TMPRSS2 (B3-TMP). Here, the selected RCL mutations were based on substrate profiling of preferred residues using a combinatorial peptide library [23], with preferred amino acids (IQFRV) substituted for residues 350 to 354 in SerpinB3, i.e., positions P4 to P1’ (IQFRV). A subsequent study combining rational ketobenzothiazole inhibitor design and multiplex substrate profiling by mass spectrometry largely confirmed these subsite preferences, with a strong preference for phenylalanine [17] indicated in the P2 position largely confirming these subsite preferences.

For a second approach, we generated a chimera with the SerpinB8 sequence of P4 to P1 replacing the SerpinB3 sequence (B3-Furin). Serpin B8 (human proteinase inhibitor 8, PI8) is an effective inhibitor of furin [24], and previous loop swap studies by Izaguirre et al. with antitrypsin defined a P6 to P1 insertion (VVRNSR) generating a chimeric antitrypsin Portland (alpha1-PDX) with a k2 for furin comparable to native SerpinB8 [25]. As the SerpinB3 RCL sequence already contains the two valine residues, we replaced the three P1 to P3 residues with the four RNSR residues. This insertion increased the RCL length to the majority superfamily consensus of 14 residues (from P1 to the hinge region residue P14 alanine), where native SerpinB3 has just 13 residues.

For two additional constructs, we initially replaced six amino acids of the RCL sequence flanking the cleavage site with seven amino acids (P7 to P1) of the unique S1/S2 cleavage site sequence of the SARS-CoV-2 spike protein. This inhibitor (B3-S_MBC_ α) is predicted to target furin, but has the potential to compete with a spike protein substrate for cleavage by any protease with activity for the multibasic site. Jaimes et al. showed that a number of proteases can cleave the spike S1/S2 site, including trypsin, matriptase, and cathepsins L and B [6]. A further SerpinB3 construct using the same (B3-S_MBC_ α) template was generated with a P5 Arginine residue (B3-S_MBC_ δ) to reflect the P681R mutation found in the S protein of the delta SARS-CoV-2 variant [26].

The recombinant SerpinB3 scaffold has advantages over both A1AT and SerpinB8; native antitrypsin has a propensity to aggregate at low pH and temperatures above 50 °C, and is predominantly found in inclusion bodies on expression of unmodified recombinant protein [27,28]. SerpinB8 has ten cysteine residues, and production of recombinant protein necessitated multiple mutations of these [25]. In contrast, SerpinB3 has one cysteine residue, and bacterial expression and purification of soluble monomeric recombinant protein is readily achieved.

### 2.2. Inhibition of Cathepsin L, TMPRSS2 and Furin by SerpinB3 Variants

**Figure 2:**
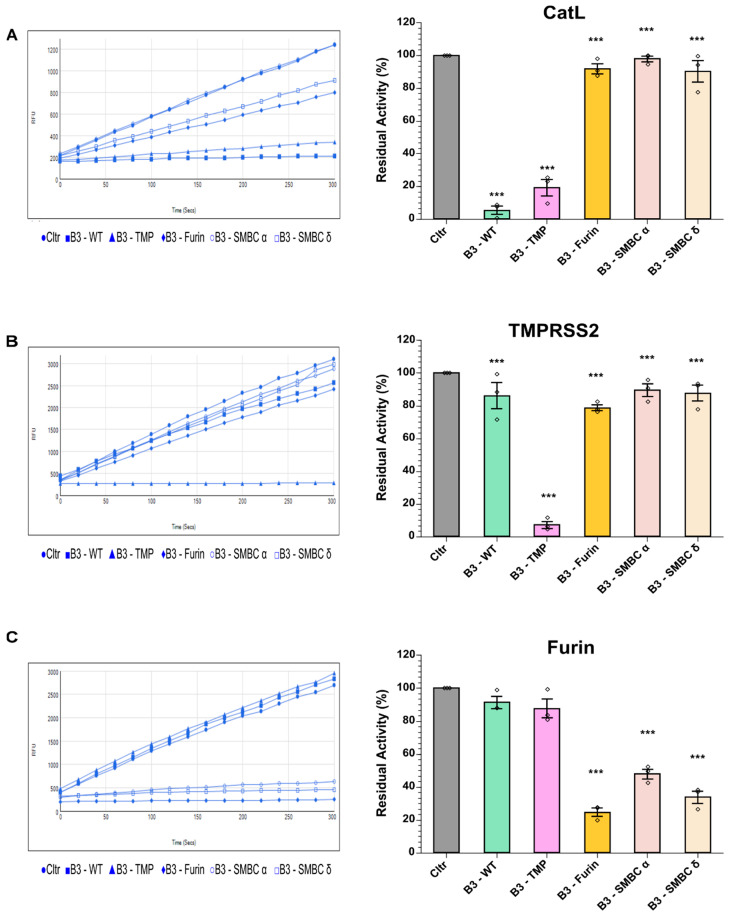
Effective change in target protease specificity of SerpinB3 RCL variants. The left panel shows raw data timecourses of protease activity (relative fluorescent units, RFU) following inhibition with purified recombinant proteins or buffer control. (**A**) CatL activity with substrate Z-FR-AMC following 2 min incubation with serpin variants, (**B**) TMPRSS-2 activity with substrate Boc-QAR-AMC following 10 min incubation with serpin variants, and (**C**) Furin residual activity with substrate Boc-RVRR-AMC following 5 min incubation with serpin variants. Residual activity is plotted with uninhibited controls taken as 100% activity. Data are shown as means ± SEM derived from n = 3. One-way ANOVA with Tukey’s comparison test: *** *p* <0.0005.

Proteins were expressed and purified by ion exchange and IMAC chromatography (Figure S1). Inhibitory activity was assessed by examining fluorometric substrate cleavage following incubation of protease with an excess of serpin. As expected, the mutations caused a loss of CatL inhibitory activity, although the B3-TMP variant retained significant CatL inhibition at 81% compared to 95% with native SerpinB3 under identical selected conditions (Figure 2A). For TMPRSS2 inhibition at a 5:1 serpin: protease ratio, the only effective variant was this construct (B3-TMP), for which 92% inhibition of TMPRSS2 was achieved (Figure 2B). For furin inhibition, the Serpin B3-B8 chimera (B3-Furin) showed greater inhibition than the two constructs containing the actual spike multibasic cleavage site residues. The variant containing the delta S cleavage sequence had marginally increased inhibition of furin over the alpha-S variant, which is consistent with the reports that the P681R mutation directly enhances the S1/S2 cleavage [29]. However, in preliminary experiments to inhibit virus infection, the spike cleavage site sequence constructs (B3-S_MBC_ α and B3-S_MBC_ δ) showed less potential to block infection, and thus these were discontinued for further studies.

### 2.3. SerpinB3 Variants Can Prevent SARS-CoV-2 Spike Degradation In Vitro

**Figure 3:**
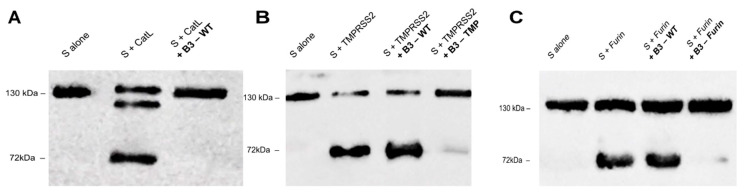
SerpinB3 variants can block cleavage of recombinant S protein by proteases in vitro. (**A**) Western blot of S protein cleavage by CatL in the presence of SerpinB3 WT after 5 h incubation (**B**) Western blot of S protein cleavage by TMPRSS2 in the presence of SerpinB3 variants (B3-WT and B3-TMP) after overnight incubation. (**C**) Western blot of S protein cleavage by furin in the presence of SerpinB3 variants (B3-WT and B3-Furin) after overnight incubation. S protein containing a C-terminal Flag tag was assessed by anti-Flag antibody. Representative data from three independent experiments are shown.

We next examined the ability of the most effective variants (WT, B3-TMP, and B3-Furin) for inhibiting cleavage of the viral substrate in vitro using recombinant SARS-CoV-2 spike protein extracellular domain (1273aa) tagged with Flag and 6XHis (S protein sequence of original Wuhan variant, P0DTC2, https://www.uniprot.org/uniprotkb/P0DTC2/entry (accessed on 19 September 2022)). Overnight incubation of S protein with both TMPRSS2 and furin or 5 h incubation with CatL resulted in cleavage with a C-terminal Flag tagged product detected by immunoblot (Figure 3).

Each specific SerpinB3 variant inhibitor was able to block S cleavage by the corresponding host protease with little or no cleavage products visible and the 135 kDa spike protein substrate remaining intact. The CatL products of approximately 105 kDa and 72 kDa suggest an additional cleavage site in the protein compared with furin and TMPRSS2. This is consistent with the recent findings of Zhao et al., who have identified novel CatL cleavage sites at positions 259 (CS-1) and 636 (CS-2) [30].

### 2.4. SerpinB3 and Modified Variants Inhibit SARS-CoV-2 Spike Mediated Pseudovirus Cell Entry

**Figure 4:**
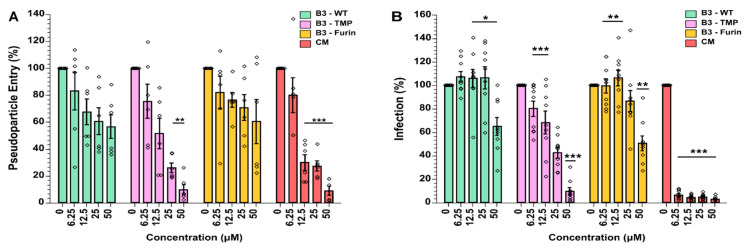
SerpinB3 variants inhibited cell entry of lentiviral S-pseudoparticles and SARS-CoV-2 infection and replication. (**A**) A549-ACE2-TMPRSS2 cells were incubated for 1 h with media (control) or with increasing concentrations of SerpinB3 variants or CM, followed by infection with SARS-CoV-2 S pseudoparticles (in 1:1 ratio). Luciferase activities (representing pseudoparticle entry) were analysed in cell lysates at 48 h post-pseudoparticle infection. (**B**) VeroE6/TMPRSS2 cells were treated with increasing concentrations of SerpinB3 variants 1 h before, simultaneously with, and 18 h post-infection with SARS-CoV-2, with the percentage of SARS-CoV-2 infected cells determined using flow cytometry to detect virus nucleocapsid staining. The mean ± SEM from n = 2 (**A**) or n = 3 (**B**) independent experiments performed in triplicates. Two-way ANOVA with Tukey’s comparison test: * *p* < 0.05, ** *p* < 0.002, *** *p* < 0.0005. CM, Camostat Mesylate.

The wild-type (anti-CatL), anti-TMPRSS2, and anti-furin variants of SerpinB3 were then brought forward to investigate their ability to prevent SARS-CoV-2 S-pseudoviral particle cell entry. Pseudoviral particles expressing S were added to A549 cells expressing ACE2 and TMPRSS2. Camostat mesylate (CM), an inhibitor of TMPRSS2, was included as a positive control. Concentrations ranging from 6.25 μM to 50 μM were tested, and luciferase activity was measured to analyze pseudoparticle entry. All three serpin constructs exhibited concentration-dependent inhibition of pseudoparticle entry, with the anti-TMPRSS2 variant (B3-TMP) having the greatest effect, showing 90% inhibition at 50 μM, which is comparable to the CM control (Figure 4A).

### 2.5. SerpinB3 Anti-TMPRSS2 Inhibited SARS-CoV-2 Infection in VeroE6-TMPRSS2 Cells

To examine the capacity of SerpinB3 variants to inhibit SARS-CoV-2 replication, we employed an infection model using Vero-E6/TMPRSS2 cells and SARS-CoV-2 (WT, D614G) where the percentage of SARS-CoV-2-infected cells was determined at 18 h post-infection (pi) by flow cytometry analysis of nucleocapsid protein-positive cells (Figure 4B). Cell viability assays showed that all SerpinB3 variants and CM were non-cytotoxic (Figure S2).

All constructs showed inhibitory activity over control. For B3-WT (inhibiting CatL) and B3-Furin (inhibiting furin), only the higher concentration (50 μM) limited SARS-CoV-2 replication by 50%. However, the construct designed to inhibit TMPRSS2 (B3-TMP) displayed good dose-dependent downregulation of viral replication, achieving up to 50% inhibition at 6.5 μM and up to 92% inhibition at 50 μM. These results are consistent with previous findings that SARS-CoV-2 entry is preferentially facilitated via membrane fusion at the cell surface following TMPRSS2-mediated Spike processing in this cellular model and less via the endosomal route and cathepsin L processing [15]. A further interpretation, considering the retention of cathepsin L inhibition in addition to the gain in TMPRSS2 inhibition, is that the B3-TMP serpin variant inhibits both pathways (Figure 2).

### 2.6. SerpinB3 Anti-TMPRSS2 (B3-TMP) Is More Effective Than A1AT at Inhibiting TMPRSS2 and Supressing SARS-CoV-2 Infection

**Figure 5:**
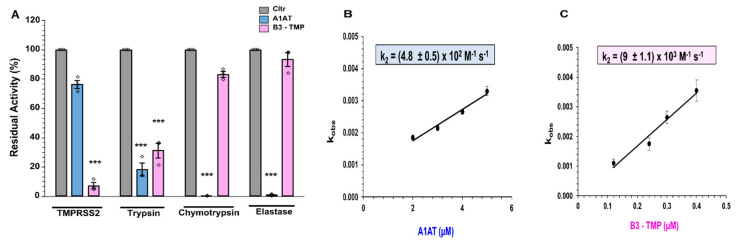
(**A**) B3-TMP variant is more effective than A1AT. B3-TMP was compared to A1AT in inhibiting TMPRSS2, trypsin, chymotrypsin, and elastase in protease assays. The means ± SE from n = 3 independent experiments. One-way ANOVA with Tukey’s comparison test: *** *p* < 0.0005. (**B**,**C**) Second order rate constant, k_2_. The residual initial velocity of substrate cleavage by TMPRSS2 (0.1 μM) was determined over time following incubation with various concentrations of (**B**) A1AT and (**C**) B3-TMP. The natural logarithm of the residual activity (ln(E)) was plotted against the time points (s) and data fitting was performed with linear regression analysis. The slopes of these lines represent -k_obs_. This k_obs_ is replot against serpin concentration to yield the second order inhibition rate constant (k_2_).

The serpin A1AT having been shown to inhibit TMPRSS2 activity and SARS-CoV-2 infection, we next made inhibitory comparisons with B3-TMP. As an indication of target protease selectivity, both serpins were tested with mammalian trypsin, chymotrypsin, and elastase, in addition to TMPRSS2. A short preincubation time of 10 min was observed prior to addition of the fluorogenic substrate, and residual activity was determined. A1AT was the more effective inhibitor of trypsin, chymotrypsin, and pancreatic elastase, but for TMPRSS2 activity B3-TMP showed 92% inhibition where A1AT exhibited 15% inhibition (Figure 5A). 

Wettstein et al. [20] reported effective inhibition of recombinant TMPRSS2 by Prolastin, a pharmaceutical preparation of A1AT from human plasma, (analysed as <62% purity [31]). In that study, residual rTMPRSS2 activity was measured over an extended 3 h incubation with serpin and substrate, in contrast to our rapid loss of activity following incubation with serpin, with real-time hydrolysis monitored. We performed a kinetic comparison, with second order rate constants of TMPRSS2 inhibition determined under pseudo-first order conditions using a range of serpin concentrations. The respective k_2_ values of (4.8 ± 0.55) × 10^2^ M^−1^ s^−1^ for A1AT (Figure 5B) and (9 ± 1.1) × 10^3^ M^−1^ s^−1^ for B3-TMP (Figure 5C) indicate an eighteen-fold increase in TMPRSS2 specificity for B3-TMP over A1AT. 

**Figure 6:**
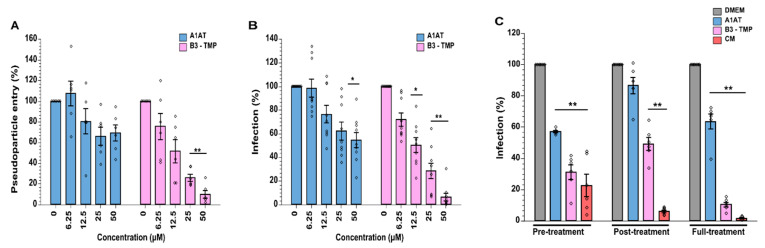
SerpinB3 anti-TMPRSS2 variant is more effective than A1AT at suppressing SARS-CoV-2 infection. (**A**) A1AT and B3-TMP were added to A549-ACE2-TMPRSS2 cells 1 h prior to infection with pseudoparticles and along with the pseudoparticles. (**B**) B3-TMP was compared with A1AT in VeroE6/TMPRSS2 cells. Cells were treated with serpin variants 1 h before, simultaneously with, and overnight after infection with SARS-CoV-2. (**C**) B3-TMP, A1AT, and CM were added as ‘Pre-treatment’ (1 h before infection and maintained for 1 h infection), ‘Post-treatment’ (added after the infection, when the virus had been removed) and ‘Full-treatment’ (both pre-treatment and then post-treatment with fresh drug) in VeroE6/TMPRSS2 cells. The mean ± SEM from n = 2 (**A**,**C**) or n = 3 (**B**) independent experiments performed in triplicates. Two-way ANOVA with Tukey’s comparison test: * *p* < 0.05, ** *p* < 0.005.

Inhibition of SARS-CoV-2 spike-mediated pseudoparticle entry was examined, and we found that the variant B3-TMP was more effective at suppressing entry of pseudoparticles in A549-ACE2-TMPRSS2 cells than A1AT (Figure 6A). Here, A1AT reached a plateau at 12.5 μM with 40% inhibition, while B3-TMP achieved 70% inhibition at 12.5 μM and 90% inhibition at 50 μM. Similarly, for SARS-CoV-2 entry into VeroE6-TMPRSS2 cells, B3-TMP was more effective at inhibiting viral entry at equivalent concentrations to A1AT (Figure 6B), with 92% inhibition at 50 μM compared to 45% for 50 μM A1AT.

To delineate how the protease inhibitors exerted their antiviral effects, we carried out a ‘Time-of-Addition’ experiment with 50 μM B3-TMP and A1AT proteins. These were added either before or at the time of viral entry (1 h prior and during 1 h of viral infection), post-treatment (18 h post-infection), or for full-time treatment during which inhibitors were added before, during infection, and post-infection for 18 h following virus removal. As seen in Figure 6C, full time treatment of B3-TMP is the more potent, with 93% inhibition of viral replication (as described above). Interestingly, B3-TMP treatment limited to viral entry remains effective to inhibit SARS-CoV-2 and achieved up to 70% of viral inhibition, strongly supporting the view that B3-TMP interferes with viral entry at all times of addition, with best results for continuous presence of serpin inhibitor. As above, A1AT displayed a similar pattern of viral inhibition, albeit less potent, indicating that A1AT inhibits viral entry as well, which is consistent with the findings of Wettstein et al. [20]. The post-entry effect suggests that our B3-TMP serpin can block new rounds of infection of progeny virus into uninfected cells. 

## 3. Discussion

The serpin superfamily of proteins has a highly conserved structure and suicide inhibitor mechanism of action [32]. Specificity for a given target protease is largely determined by the sequence of the exposed RCL, raising the potential for designer serpins with inhibitory potential for proteases with no known serpin inhibitor, or of generating a more selective serpin that may have multiple physiological targets. The concept of designer serpins dates back to the naturally occurring A1AT-Pittsburg mutation, where a P1 mutation to arginine (M358R) generated a hemorrhagic disease causing antithrombin activity in patient plasma [33]. Other cleavage site alterations have been deliberately performed to change specificities of A1AT–Pittsburg [34], and prior studies with loop swaps demonstrate that the inhibitory activity can be interchangeable between serpin scaffolds [25,35]. Polderdijk et al. designed a more specific APC serpin inhibitor using a structural approach to optimize P2 to P1’ residues in A1AT- Pittsburg, generating a potent inhibitor [36]. 

Such detailed structural data did not exist for TMPRSS2 until recently [37]. Here, we have used data from the substrate peptide library of Lucas et al. to mutate RCL residues in SerpinB3 [23]. The success of this approach, as seen in Figure 2B, was not a certainty, and previous attempts using this approach for A1AT have reported poor translation from such libraries [38]; there is a possibility that the modified serpin will result in the kinetic pathway favoring the substrate route rather than a complex formation inhibitor pathway. This has been explored using a Conserpin, a synthetic consensus serpin scaffold [39]. To our knowledge this is the first successful example of a designer serpin solely based on substrate peptide library data, and the availability of this methodology [40] could be investigated more extensively for novel inhibitor generation. Random mutagenesis of RCL residues and the development of phage display serpin libraries [41] may ultimately yield powerful novel inhibitors, although for both serpin expression and target protease availability this approach can be constrained due to high-throughput screening limitations.

At the outset, our rationale was to have a common serpin scaffold for a range of host protease targets, with the future potential to use a combination of these proteins for maximal inhibitory effect. For an in vivo human or animal infection, the virus will encounter and potentially infect a number of cell types, some lacking TMPRSS2 expression [42]. The SerpinB3 scaffold has the advantages of known ability to target both cysteine and serine proteases, increased thermostability in comparison with A1AT, and easier recombinant protein production in comparison with SerpinB8 due to the latter containing ten cysteine residues. 

While a combination therapy targeting host proteases may prove favorable, there is a strong case that TMPRSS2 alone is the most attractive target for SARS-CoV-2 variants of concern that cause severe disease [18]. Furin is an essential protease for numerous protein processing events [43], and a CatL-deficient mouse exhibits abnormal skin hair and bone homeostasis, with inhibitory drugs predicted to impair the human adaptive immune system [44]. However, a TMPRSS2 null mouse displayed no negative phenotype [45], and although expressed in human prostate, lung, and other epithelial cells, a normal biological function for this enzyme remains unclear. While the enzyme is able to activate pro-HGF, this activity is shared with other type II transmembrane serine proteases. In addition to viral infection, TMPRSS2 is a potential target for cancer therapy [46] with well-documented links to prostate cancer metastasis, where a *TMPRSS2–ERG* fusion gene is found in 50% of prostate tumours [47].

Based on findings that A1AT can inhibit TMPRSS2, a repurposing of approved therapeutics such as Prolastin for SARS-CoV-2 infection has been proposed [20] and entered a number of clinical trials for COVID-19. However wild-type A1AT is a potent inhibitor of neutrophil elastase, pancreatic elastase, trypsin, chymotrypsin, tissue kallikrein 7 and 14, cathepsin G, and proteinase 3 [48], making unwanted off-targets a likely consequence. As discussed above, the reported TMPRSS2 inhibition by antitrypsin was determined by fluorometric substrate hydrolysis following 3 h incubation with substrate. Here, we observe 92% loss of TMPRSS2 activity following 10 min incubation with the modified serpin B3-TMP, a time frame consistent with the rapid suicide mechanism of serpins. The B3-TMP showed better selectivity for TMPRSS2 (Figure 5A), and kinetic comparisons demonstrate that B3-TMP has a second order rate constant for TMPRSS2 inhibition that is eighteen-fold greater than A1AT (Figure 5B,C). 

Recent studies on the Omicron variants of SARS-CoV-2 report an entry route less dependent on TMPRSS2 spike cleavage and reduced ability to induce syncytia in tissue culture, which may explain less efficient replication in lung epithelial cells. Findings suggest that Omicron variants may preferentially use the endosomal entry route; for the lineage BA.1, Calu-3 cell entry showed lower sensitivity to Camostat and increased sensitivity to the cathepsin inhibitor E-64d when compared with the Delta variant [49]. Thus, while the native SerpinB3 cathepsin L inhibitor may have the ability to block Omicron variants, a noteworthy result here is that our B3-TMP variant is an effective inhibitor of TMPRSS2 while retaining significant inhibition of CatL (Figure 2A), suggesting a potential dual-target effect to suppress cell entry.

As naturally occurring inhibitors present in plasma and other tissues at concentrations up to 40 μM, serpins have low immunogenicity and toxicity, and with minor RCL residue changes as shown here, designer serpins are strong candidates for evaluation in viral infection. Unlike therapeutics that block the specific viral-encoded main M-Pro protease, inhibitors of host cell surface proteases may have broader antiviral potential for both influenza and coronavirus infections. A further possibility is synergistic use of serpin host protease inhibitors in combination with inhibitors of virally encoded targets to reduce the potential for drug resistance and improve disease outcomes.

## 4. Materials and Methods

### 4.1. Mutagenesis of SerpinB3

The full-length open reading frame cDNAs for SerpinB3 (SCCA-1) had previously been cloned into the pRSETC (Invitrogen, Dublin, Ireland))expression vector [50]. Using this plasmid containing wild-type sequence (GenBank: AB046399.1) as a template, RCL alterations were performed using Quikchange^®^ Site Directed Mutagenesis, where primers were designed with extensions up to 20 nucleotides to replace RCL residues (Supplementary Table S1). PCR products were Dpn1-treated and initially transformed into DH5α compètent cells. Following sequence verification (Eurofins Genomics, Ebersberg, Germany), plasmids were transformed into *E. coli* BL21(DE3) cells (NEB).

### 4.2. Expression and Purification of Recombinant Proteins

Transformed *E. coli* BL21(DE3) cells were grown in Overnight Express autoinducing medium (Formedium) containing 100 μg/mL ampicillin for 16 h at 37 °C. Cells were harvested by centrifugation at 4000× *g* for 20 min and lysed using B-PER lysis reagent (Thermo Fisher Scientific, Frederick, MD, USA)). Soluble material was clarified by centrifugation of the lysate at 12,500× *g* for 20 min at 4 °C, followed by 0.22 μm filtration. The filtrate was applied to Q-Sepharose (Sigma Aldrich Ltd., Dublin, Ireland)) resin equilibrated with 50 mM Tris-HCl pH 7.0 as a negative ion-exchange step to remove *E. coli* proteins. The unbound fraction was applied to Chelating Sepharose Fast Flow resin (GE Healthcare Bio-Science AB, Uppsala, Sweden) pre-charged with 50 mM NiSO_4_ and equilibrated with 150 mM NaCl and 50 mM Tris-HCl, pH 7.9. Following five column volume washes with this buffer containing 20 mM imidazole, proteins were eluted with 0.5 M imidazole and buffer was exchanged into either PBS or DMEM for further analysis. Prior to cell infection experiments, endotoxins were removed using Endotoxin removal columns (Thermo Fisher Scientific, Frederick, MD, USA) according to the manufacturer’s instructions.

### 4.3. Protease Inhibition Assays 

The protease specificity of purified proteins was assessed by monitoring the linear rate of cleavage of fluorogenic substrates. Cathepsin L was gifted by Dr Matej Vizovišek, Jozef Stefan Institute, Slovenia. Recombinant human TMPRSS2 was obtained from Cusabio Technology, Houston, TX, USA) (CSB-YP023924HU) and recombinant human furin from BioLegend, CA, USA (719404).

Proteases were incubated with a 1:5 molar excess of serpin and residual activity was determined by cleavage of fluorescent substrates. All assays were carried out at room temperature in 96-well plates in a total volume of 100 µL. Residual proteolytic activity was measured using a SpectraMax M3 Microplate Reader (Molecular Devices, Inc., San Jose, CA, USA) using the appropriate buffer and synthetic fluorogenic peptide substrate: Cathepsin L, Z-FR-AMC (10 μM), 50 mM Na-Acetate, 100 mM NaCl, 1 mM EDTA, pH 5.5; Chymotrypsin, Z-AAPF-AMC (5 μM), PBS, 1 mg/mL BSA; Elastase, Met-O-Suc-AAPV-AMC (10 μM), 50 mM Tris, 120 mM NaCl, pH 8.0, 1 mg/mL BSA; Furin, Boc-RVRR-AMC (50 μM) 20 mM Tris, 1 mM CaCl2, 0.5% Brij-35, pH 9; TMPRSS2, Boc-QAR-AMC (80 μM), PBS; Trypsin, Z-FR-AMC (10 μM), 50 mM Tris, 0.2 mM DTT, 1 mg/mL BSA. The excitation and emission wavelengths used were 380 nm and 460 nm, respectively. All assays were performed in triplicate.

For kinetic analysis of TMPRSS2 inhibition, second order inhibition rate constants were determined under pseudo-first order conditions using a range of serpin concentrations and with the TMPRSS2 buffer and substrate detailed above. Reactions were performed at room temperature and residual activity was determined at intervals over 10 min incubation with a molar excess of serpin over protease. Concentration ranges were 0.12 to 0.44 μM for B3-TMP and 1.0 to 4 μM for A1AT. The apparent first-order rate constant k_obs_ for each concentration was calculated from the slope of the natural log of residual activity over time (-k_obs_). These values were plotted against inhibitor concentration, and linear regression analysis of this plot provided the second-order inhibition rate constant k_2_ with the standard error determined.

### 4.4. Spike Protein Cleavage 

Recombinant spike protein UniProt ID P0DTC2 with both His and Flag Tag (GenScript, Z03481) consisted of the extra cellular domain (ECD) with the sequence Val16-Pro1213 fused to poly His and Flag tags at the C-terminus. S protein in both the presence and absence of purified recombinant serpin was incubated with either furin, TMPRSS2, or CatL in the appropriate assay buffer, as above. After 24 h (or 5 h for CatL cleavage), products were analysed by immunoblot with monoclonal anti-FLAG antibody (1:2000; Merck, Rahway, NJ, USA). The protein band was visualized using a Vilber Fusion imaging system following incubation with anti-mouse IgG HRP-linked secondary antibody (1:5000; Cell Signaling; 7076S).

### 4.5. Cell Culture

A549-ACE2-TMPRSS2 cells (gifted from Prof. Suzannah J Rihn) were maintained as described [51]. Vero-E6/TMPRSS2 cells (#100978) were obtained from the Centre for AIDS Reagents (CFAR), National Institute for Biological Standards and Control (NIBSC) [13]. Cells were cultured in Dulbecco’s modified Eagle medium (DMEM Thermo Scientific, 61965-026) supplemented with GlutaMAX™ and 10% (DMEM-10) or 2% (DMEM-2) foetal bovine serum (FBS) (Themo Scientific, 10500-064) and Geneticin (Thermo Scientific, 10131035) at a concentration of 1 mg/mL, at 37 °C in 5% CO_2_. Cells routinely tested negative for mycoplasma.

### 4.6. Generation of Lentiviral Pseudoparticles

Either SARS-CoV-2 Spike pseudoparticles (Spp) or no-glycoprotein (NE) pseudoparticle controls were produced in HEK293T cells [52]. Cells were seeded in 100 mm dishes at 4 × 10^6^/well 24 h prior to transient transfection with three plasmids; 3.545 μg P8.91 (encoding for HIV-1 gag-pol), 3.545 μg CSFLW (lentivirus backbone expressing a firefly luciferase reporter gene), and 150 ng of either SARS-CoV-2 Spike (Accession No. MN908947.3, with Spike coding sequence cloned in pcDNA3.1) or NE (empty plasmid, pcDNA3.1). Transfected cells were incubated at 37 °C, 5% CO_2_. After 24 h, the transfection mix was replaced with DMEM containing 10% (v/v) FBS. Harvested supernatants containing Spp or NE pseudoparticles were taken at 48 h and 72 h post-transfection, pooled, and centrifuged at 1200× *g* for 10 min at 4 °C to remove cellular debris, then aliquoted and stored at −80 °C. 

### 4.7. Assay for Pseudoparticle Cell Entry Inhibition

A549-ACE2-TMPRSS2 cells were seeded in 96-well plates at 3.75 × 10^4^/well one day prior to treatment and transduction. Prior to transduction with Spp or NE, cells were treated with 100 µL of serpin variants for 1 h, then media were removed and replaced with 200 µL of either Spp or NE mixed 1:1 with the serpin variants and cells were incubated for 48 h at 37 °C, 5% CO_2_. To quantify firefly luciferase, cells were lysed in 50 µL/well Passive Lysis Buffer (Promega, NY, USA) and cell lysates were analyzed for luciferase activity using a luminometer, then the firefly luciferase activity produced by Spp was normalised to NE control. SerpinB3 variants were tested in three technical replicates and two independent experiments.

### 4.8. SARS-CoV-2 Clinical Isolates 

Live SARS-CoV-2 experiments were carried out in a Containment Level 3 laboratory under Biosafety Level 3 guidelines. WT clinical isolate with D614G substitution (CEPHR_IE_B.177.18_1220, GenBank accession ON350866, Passage 1) was isolated on Vero-E6/TMPRSS2 cells from SARS-CoV-2 positive nasopharyngeal swabs. Viral RNA was isolated using a Qiagen Viral mRNA mini kit according to the manufacturer’s instructions. Viral RNA genome was sequenced to confirm the integrity of the Spike protein. The titres of the virus stocks were determined by plaque assay. WT (D641G) had a titre of 2.6 × 10^6^ Plaque Forming Units (PFU) per mL. 

### 4.9. VeroE6-TMPRSS2 Viral Infection Assay

Vero-E6/TMPRSS2 cells were plated (2.5 × 10^4^ cells per well) in 96-well clear flat-bottom plates and incubated overnight at 37 °C 5% CO_2_ to reach 90–100% confluency at time of infection. The Drug Dose Response of SerpinB3 variants, alpha-1 antitrypsin (A1AT), and camostat mesylate (CM) were performed using four points and two-fold dilutions from 50 µM stock, as indicated. Following 1 h pre-treatment with drugs in DMEM-2 at 37 °C 5% CO_2_, WT (D614G) SARS-CoV-2 (520 PFU per well of 2.5 × 10^4^ cells for a Multiplicity of Infection (MOI) of 0.02) was added to the cells in drug-containing DMEM-2 for 1 h at 37 °C 5% CO_2_. Cells were then washed twice in dPBS (Thermo Scientific, 14190094) and further incubated in DMEM-2 containing drugs for 18 h at 37 °C 5% CO_2_. Drugs were tested in technical triplicate with a minimum of three independent experiments.

For time-of-addition experiments, cells were exposed to protease inhibitors (50 µM) or control in three drug addiction schemes run concurrently. “Pre-treatment”: drug treatment was limited to 1 h pre-treatment and 1 h during viral exposure. “Post-treatment”: following dPBS washes, drugs were added immediately post-infection and for 18 h. “Full-treatment”: both pre-treatment and then post-treatment, as described above, were included. Drugs were tested in three technical replicates and two independent experiments.

### 4.10. Flow Cytometry Analysis of SARS-CoV-2 Infected Cells 

At 18 h post-infection, cells were washed in dPBS and trypsinised (Trypsin-EDTA, Thermo Scientific, 15400054) to achieve single cell suspension. Cells were fixed in 4% formaldehyde solution (Sigma Aldrich Ltd., Dublin, Ireland, F8775) in the dark at room temperature for a minimum of 8 h. All steps-post fixation were carried out in Biosafety Level 2 conditions in Class 2 Biosafety Cabinets. Cells were permeabilised with Perm/Wash Buffer (BD, 554723) according to the manufacturer’s instructions and this was maintained throughout antibody staining. Intracellular SARS-CoV-2 Nucleoprotein (NP) staining was performed with SARS/SARS-CoV-2 Nucleocapsid Monoclonal Antibody (E16C) (1/100 dilution, Invitrogen, MA1-7403) and goat anti-mouse IgG2b-FITC (1/500 dilution, Santa Cruz Biotechnology, Texas, USASC-2080). Cells were resuspended in 60 µL PBS-EDTA-2% (Sigma-Aldrich, 03690-100 mL) for flow cytometry analysis (CytoFlex S, Beckman Coulter, Pasadena, CA, USA). Cells were gated with Forward and Side-Scatter gates to exclude debris from intact cells, then single cells were gated based on linearity between Area and Height. The percentage of infected cells analysed using the FITC-channel (Blue 488 nM laser, 525/40 laser). Analysis was performed using CytExpert software (version 2.4.0.28, Beckman Coulter, Pasadena, CA, USA). The mean percentage of infected cells was determined from the technical replicates and normalised to the positive (virus alone) and negative (DMEM-2) controls to determine the percentage of viral inhibition.

### 4.11. Cell Viability Assay

Vero-E6/TMPRSS2 cells were plated (2.5 × 10^4^ cells per well) in 96-well clear flat-bottom plates and incubated overnight at 37 °C 5% CO_2_ to reach 90–100% confluency at time of infection. Dilution of serpin variants, A1AT, and CM were performed using four points and two-fold dilutions from 50 µM stock, as indicated. Cells were pre-treated with 100 µL drugs for 2 h at 37 °C 5% CO_2_. 100 µL DMEM-2 was then added to the wells and cells were incubated overnight at 37 °C 5% CO_2_. Cell death was forced in triplicate wells using by incubating cells in 20% DMSO solution for 10 min at 37 °C 5% CO_2_. Supernatant was removed and cells were washed twice in dPBS before staining with Viobility™ 405/452 Fixable Dye (130-130, 1/100 dilution, Miltenyi Biotech, Westphalia, Germany) according to the manufacturer’s instructions. Cells were washed twice in dPBS, trypsinised, and fixed in 4% formaldehyde solution. Cells were then resuspended in 60 µL PBS-EDTA-2% for flow cytometry analysis (CytoFlex S, Beckman Coulter, Pasadena, CA, USA). Cells were gated with Forward and Side-Scatter gates to exclude debris from intact cells, then single cells were gated based on linearity between area and height. Viobility-stained cells were detected in the PB450 channel (Violet 405 nM laser, 450/45 filter). A negative control (DMEM alone) was used to set the boundary of the live cells, while a positive control (DMSO-treated) was used to ensure uptake of the stain by dead cells. Analyses were performed using CytExpert software (version 2.4.0.28, Beckman Coulter). The mean percentage of dead cells was determined from the technical replicates and presented as the percentage of viable cells per condition (100% dead cells).

### 4.12. Statistical Analysis 

All statistical analyses were performed using Minitab version 18. For statistical analysis, either ordinary one-way ANOVA or two-way ANOVA with Tukey’s comparison test was used, as indicated in figure legends.

## Data Availability

Datasets generated and analysed during the study are available from the corresponding author upon reasonable request.

## Supplementary Materials

The following supporting information can be downloaded at: https://www.mdpi.com/article/10.3390/ijms232012522/s1.

## Abbreviations

A1AT	Alpha-1 antitrypsin
ACE2	Angiotensin-converting enzyme 2
CM	Camostat mesylate
CatL	Cathepsin L
COVID-19	Coronavirus disease 2019
IMAC	Immobilized metal affinity chromatography
k_2_	Second order inhibition rate constant
NE	No-glycoprotein, no-enveloped pseudoparticle control
RCL	Reactive centre loop
SARS-CoV-2	Severe acute respiratory syndrome coronavirus 2
SDM	Site-directed mutagenesis
SERPIN	Serine protease inhibitor
S	Spike protein
Spp	Spike pseudoparticles
TMPRSS2	Transmembrane protease serine 2
WT	Wild type
