# Supplementary material for: Reactive Centre Loop Mutagenesis of SerpinB3 to Target TMPRSS2 and Furin: Inhibition of SARS-CoV-2 Cell Entry and Replication
Source: Int J Mol Sci. 2022 Oct 19;23(20):12522. doi: 10.3390/ijms232012522 (PMC9604144; doi:10.3390/ijms232012522)
Supplement: Supplementary file 1 [file ijms-23-12522-s001.zip › Supplementary Materials.pdf]

Supplementary Materials

Table S1- Primer sequence for generation of SerpinB3 Variants

Variant		Sequence
**B3 - TMP**	Forward	5′ atc caa ttt aga gtc tca cct gct tca act aat gaa 3′
	Reverse	5′ gac tct aaa ttg gat tac agc ggt ggc agc tgc agc 3′
**B3 - Furin**	Forward	5′ gta cgc aat tca cgc tca tca cct gct tca act aat gaa 3′
	Reverse	5′ tga gcg tga att gcg tac tac agc ggt ggc agc tgc agc 3′
**B3 - S_{MBC} α**	Forward	5 ′ aac agc ccg cgc cgc gcc cgc tca tca cct gct tca act 3 ′
	Reverse	5 ′ gcg ggc gcg gcg cgg gct gtt ggt ggc agc tgc agc ttc 3 ′
**B3 - S_{MBC} δ**	Forward	5 ′ gct gcc acc aac agc cgg cgc cgc gcc cgc tca 3 ′
	Reverse	5 ′ tga gcg ggc gcg gcg ccg gct gtt ggt ggc agc 3 ′

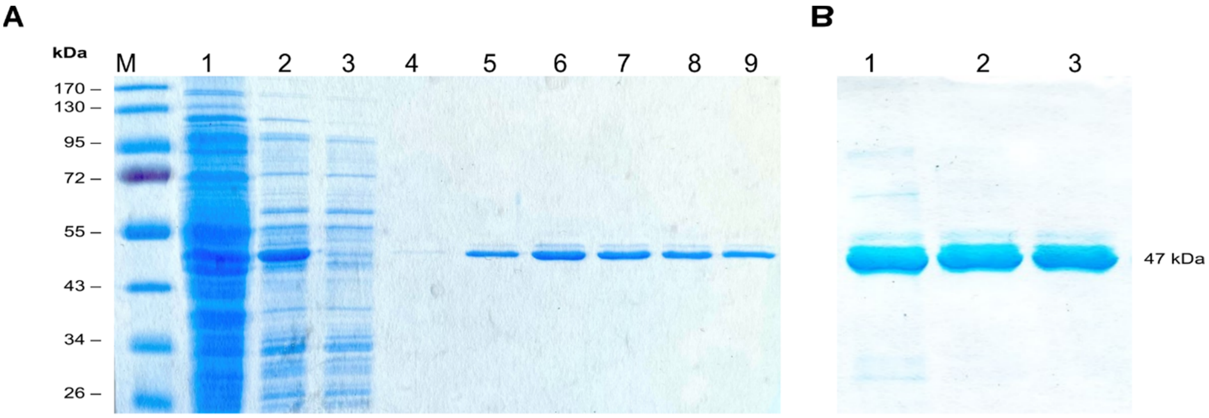

Figure S1. (A) Ion exchange and IMAC elution profiles of soluble fraction of SerpinB3 WT. Lane **M** = pre-stained protein marker, Lane**1** = Filtered fraction, Lane**2** = Unbound fraction after Ion exchange at pH 7.0, Lane**3** = Unbound fraction after IMAC, Lane**4** = Wash fraction, Lane**5-9** = Eluted fractions, **(B) Concentrated and desalted SerpinB3 variants for cell infection studies**. Lane**1** = B3-WT, Lane**2** = B3-TMP, Lane**3** = B3-Furin.

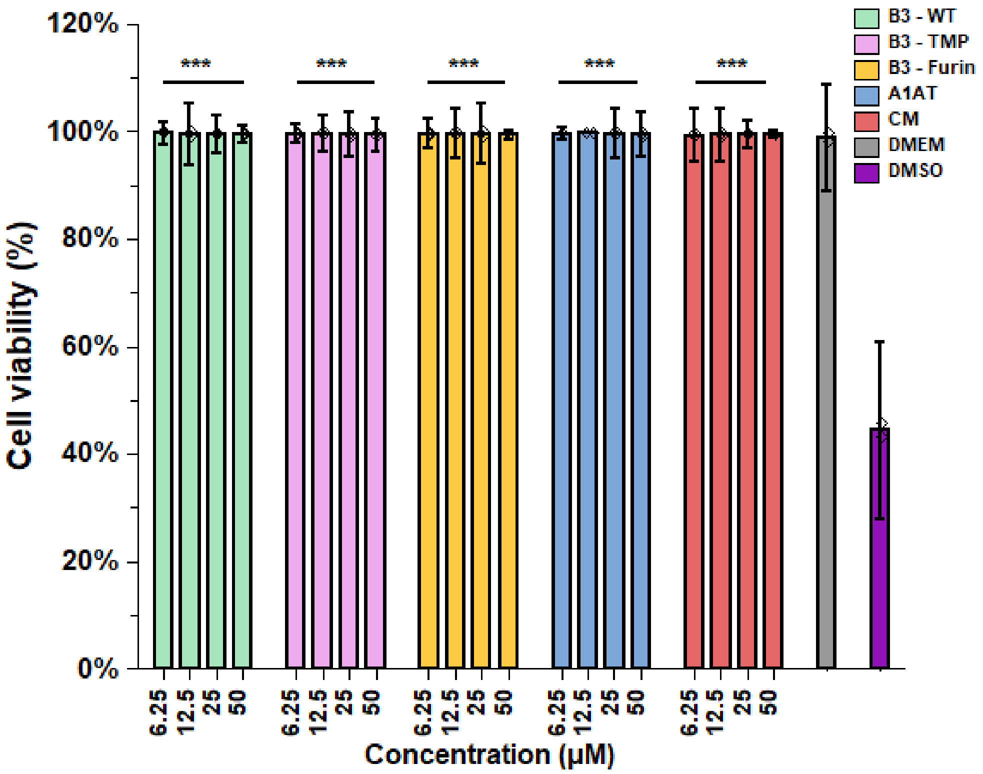

Figure S2. Cell Viability assay of SerpinB3 variants in VeroE6-TMPRSS2 expressing cells. VeroE6-TMPRSS2 expressing cells were assessed according for their viability after 18 h at the indicated concentrations of Serpin variants, A1AT or CM as described in Section 4.11. DMSO (20%) was used to induce death as a positive control. Results are presented as the mean ± SE of 2 independent experiments performed in 6 replicates. 2-way ANOVA with Tukey's comparison test: *** P <0.0005. A1AT, alpha-1 antitrypsin. CM, Camostat mesylate.
